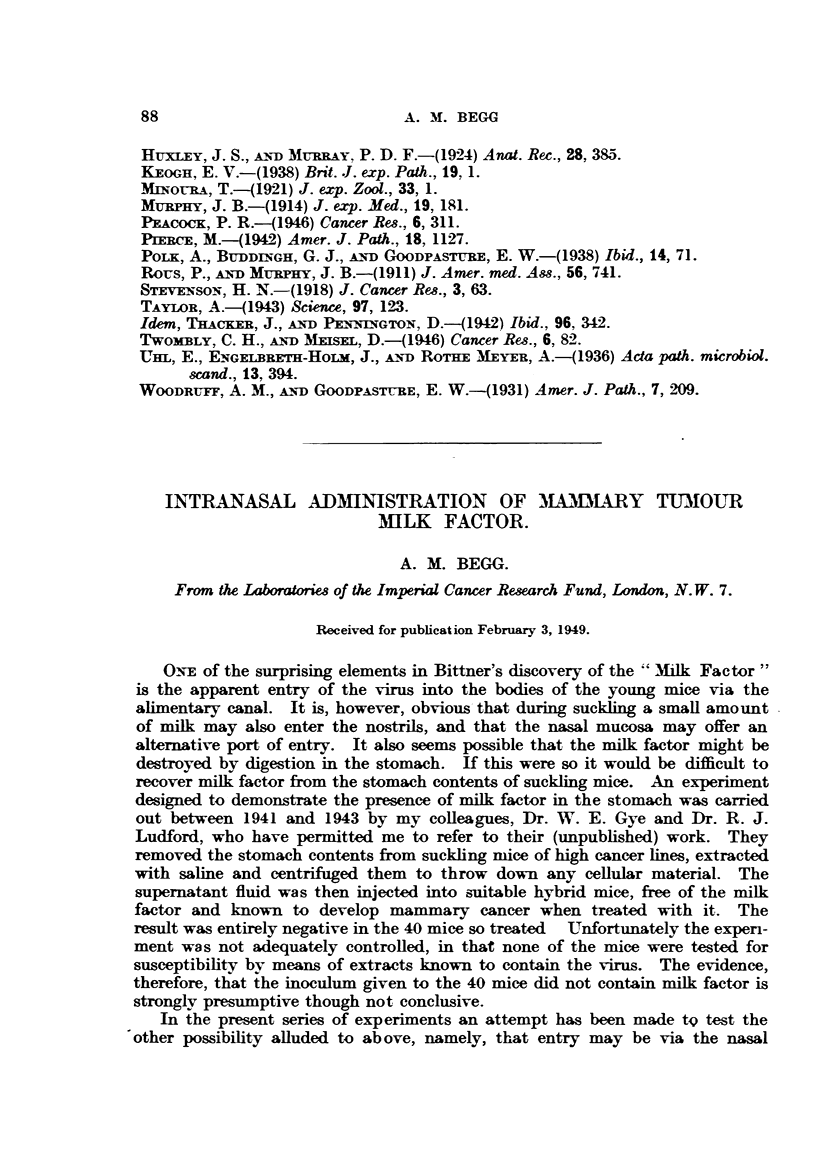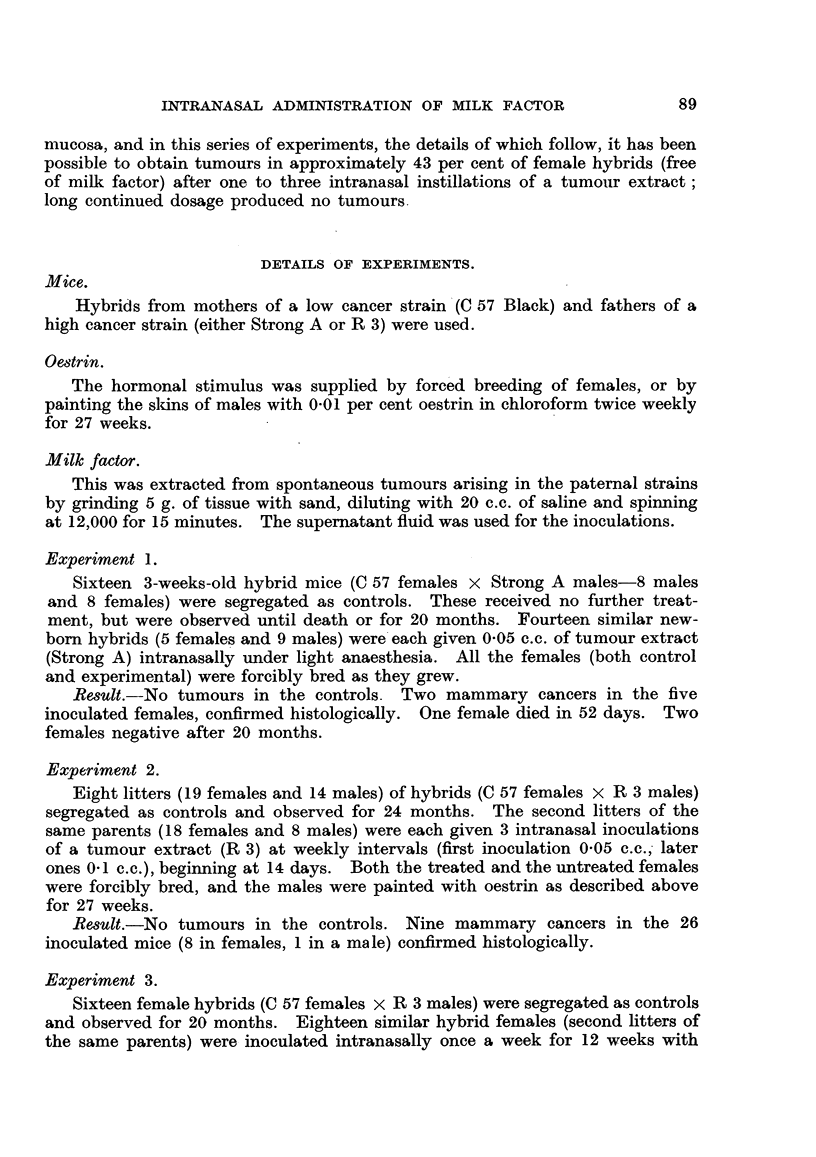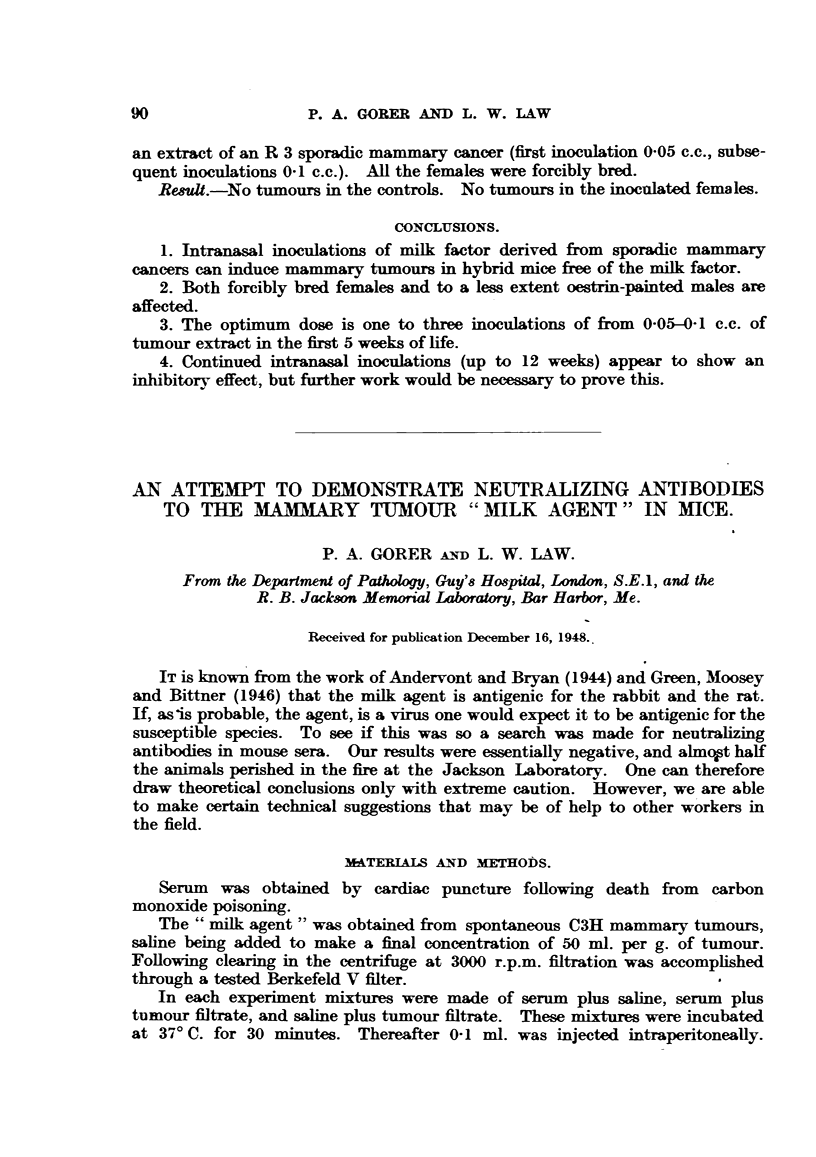# Intranasal Administration of Mammary Tumour Milk Factor

**DOI:** 10.1038/bjc.1949.9

**Published:** 1949-03

**Authors:** A. M. Begg


### INTRANASAL ADMINISTRATION OF MAMMARY TUMOUR MILK FACTOR.

A. M. BEGG.

*From the Laboratories of the Imperial Cancer Research Fund, London, N.W. 7.*

Received for publication February 3, 1949.

ONE of the surprising elements in Bittner's discovery of the " Milk Factor " is the apparent entry of the virus into the bodies of the young mice via the alimentary canal. It is, however, obvious that during suckling a small amount of milk may also enter the nostrils, and that the nasal mucosa may offer an alternative port of entry. It also seems possible that the milk factor might be destroyed by digestion in the stomach. If this were so it would be difficult to recover milk factor from the stomach contents of suckling mice. An experiment designed to demonstrate the presence of milk factor in the stomach was carried out between 1941 and 1943 by my colleagues, Dr. W. E. Gye and Dr. R. J. Ludford, who have permitted me to refer to their (unpublished) work. They removed the stomach contents from suckling mice of high cancer lines, extracted with saline and centrifuged them to throw down any cellular material. The supernatant fluid was then injected into suitable hybrid mice, free of the milk factor and known to develop mammary cancer when treated with it. The result was entirely negative in the 40 mice so treated Unfortunately the experiment was not adequately controlled, in that none of the mice were tested for susceptibility by means of extracts known to contain the virus. The evidence, therefore, that the inoculum given to the 40 mice did not contain milk factor is strongly presumptive though not conclusive.

In the present series of experiments an attempt has been made to test the other possibility alluded to above, namely, that entry may be via the nasal mucosa, and in this series of experiments, the details of which follow, it has been possible to obtain tumours in approximately 43 per cent of female hybrids (free of milk factor) after one to three intranasal instillations of a tumour extract; long continued dosage produced no tumours.

### DETAILS OF EXPERIMENTS.

*Mice.*

Hybrids from mothers of a low cancer strain (C 57 Black) and fathers of a high cancer strain (either Strong A or R 3) were used.

*Oestrin.*

The hormonal stimulus was supplied by forced breeding of females, or by painting the skins of males with 0·01 per cent oestrin in chloroform twice weekly for 27 weeks.

*Milk factor.*

This was extracted from spontaneous tumours arising in the paternal strains by grinding 5 g. of tissue with sand, diluting with 20 c.c. of saline and spinning at 12,000 for 15 minutes. The supernatant fluid was used for the inoculations.

*Experiment* 1.

Sixteen 3-weeks-old hybrid mice (C 57 females × Strong A males—8 males and 8 females) were segregated as controls. These received no further treatment, but were observed until death or for 20 months. Fourteen similar newborn hybrids (5 females and 9 males) were each given 0·05 c.c. of tumour extract (Strong A) intranasally under light anaesthesia. All the females (both control and experimental) were forcibly bred as they grew.

*Result.*—No tumours in the controls. Two mammary cancers in the five inoculated females, confirmed histologically. One female died in 52 days. Two females negative after 20 months.

*Experiment* 2.

Eight litters (19 females and 14 males) of hybrids (C 57 females × R 3 males) segregated as controls and observed for 24 months. The second litters of the same parents (18 females and 8 males) were each given 3 intranasal inoculations of a tumour extract (R 3) at weekly intervals (first inoculation 0·05 c.c., later ones 0·1 c.c.), beginning at 14 days. Both the treated and the untreated females were forcibly bred, and the males were painted with oestrin as described above for 27 weeks.

*Result.*—No tumours in the controls. Nine mammary cancers in the 26 inoculated mice (8 in females, 1 in a male) confirmed histologically.

*Experiment* 3.

Sixteen female hybrids (C 57 females × R 3 males) were segregated as controls and observed for 20 months. Eighteen similar hybrid females (second litters of the same parents) were inoculated intranasally once a week for 12 weeks with an extract of an R 3 sporadic mammary cancer (first inoculation 0·05 c.c., subsequent inoculations 0·1 c.c.). All the females were forcibly bred.

*Result.*—No tumours in the controls. No tumours in the inoculated females.

### CONCLUSIONS.

1. Intranasal inoculations of milk factor derived from sporadic mammary cancers can induce mammary tumours in hybrid mice free of the milk factor.
2. Both forcibly bred females and to a less extent oestrin-painted males are affected.
3. The optimum dose is one to three inoculations of from 0·05–0·1 c.c. of tumour extract in the first 5 weeks of life.
4. Continued intranasal inoculations (up to 12 weeks) appear to show an inhibitory effect, but further work would be necessary to prove this.